# Effect of the Carbon Concentration, Blend Concentration, and Renewal Rate in the Growth Kinetic of *Chlorella* sp.

**DOI:** 10.1155/2014/205184

**Published:** 2014-12-15

**Authors:** Adriano Arruda Henrard, Gabriel Martins da Rosa, Luiza Moraes, Michele Greque de Morais, Jorge Alberto Vieira Costa

**Affiliations:** ^1^Laboratory of Biochemical Engineering, College of Chemistry and Food Engineering, Federal University of Rio Grande, Italy Avenue on km 8th, P.O. Box 474, 96203-900 Rio Grande, RS, Brazil; ^2^Laboratory of Microbiology and Biochemical, College of Chemistry and Food Engineering, Federal University of Rio Grande, Italy Avenue on km 8th, P.O. Box 474, 96203-900 Rio Grande, RS, Brazil

## Abstract

The microalgae cultivation can be used as alternative sources of food, in agriculture, residual water treatment, and biofuels production. Semicontinuous cultivation is little studied but is more cost-effective than the discontinuous (batch) cultivation. In the semicontinuous cultivation, the microalga is maintained in better concentration of nutrients and the photoinhibition by excessive cell is reduced. Thus, biomass productivity and biocompounds of interest, such as lipid productivity, may be higher than in batch cultivation. The objective of this study was to examine the influence of blend concentration, medium renewal rate, and concentration of sodium bicarbonate on the growth of *Chlorella* sp. during semicontinuous cultivation. The cultivation was carried out in Raceway type bioreactors of 6 L, for 40 d at 30°C, 41.6 *µ*mol m^−2^ s^−1^, and a 12 h light/dark photoperiod. Maximum specific growth rate (0.149 d^−1^) and generating biomass (2.89 g L^−1^) were obtained when the blend concentration was 0.80 g L^−1^, the medium renewal rate was 40%, and NaHCO_3_ was 1.60 g L^−1^. The average productivity (0.091 g L^−1^ d^−1^) was achieved with 0.8 g L^−1^ of blend concentration and NaHCO_3_ concentration of 1.6 g L^−1^, independent of the medium renewal rate.

## 1. Introduction

The large-scale commercial microalgae cultivation began in the 1960s in Japan with cultivation of* Chlorella*, followed in the 70s–90s by the cultivation of* Spirulina* in Mexico, in USA, and in China. A common feature of most species of microalgae produced commercially (*Chlorella*,* Spirulina*, and* Dunaliella*) is that they grow in selective media and can be grown in open cultivation and still remain relatively free of contamination by other microorganisms [1].

The microalga* Chlorella* has GRAS (Generally Recognized as Safe) certification issued by the FDA (Food and Drug Administration) and can be used as food with no risk to health [2]. Microalgal biomass produced can be exploited as a source of biocompounds with high nutritional and commercial value, such as proteins, fatty acids, vitamins, pigments, and biofuels, or in the formulation of foods and animal feed [3].

Through the years, there is increasing interest in the mass culturing of microalgae for the production of biofuel as well as for use in the pharmaceutical industry, agrochemicals, and animal feed, whereas ways to increasing biomass productivity need to be more investigated and studied to improve commercial viability [4].

Cultivation conditions considerably influence the composition of microalgae. Several factors can influence the cultivation of algae in both open cultivation and closed cultivation such as pH, light, contaminants, temperature, bioreactor type, and the initial concentration of biomass [5–8]. The mode of operation is directly related to the biotechnological process, because the microorganism needs appropriate conditions to stimulate the synthesis of the desired product.

The semicontinuous cultivation is an operation mode wherein the bioreactor is loaded with culture medium at the beginning and the microorganism is maintained in optimum conditions, which are predetermined. After a certain period of time, a portion of the culture medium with the microalgae is removed and replaced with fresh culture medium without cells [9]. This type of cultivation has operational advantages, such as dispensing with the constant changing of the inoculums and allowing the microorganism to grow at a high speed [10]. Although widely used, this system of cultivation has hardly been studied, and there is a lack of scientific work involving variables such as blend concentration and medium renewal rate.

The work aims to study the* Chlorella* sp. microalga semicontinuous cultivation in Raceway type bioreactor, assessing the influence of the blend concentration, medium renewal rate, and concentration of sodium bicarbonate on the growth kinetics and on the produced biomass of microalga.

## 2. Materials and Methods

### 2.1. Microorganism and Culture Medium

The microalga used in this study was* Chlorella* sp., maintained and cultivated in BG-11 medium [11], containing (g L^−1^) NaNO_3_ (1.50); K_2_HPO_4_ *·*3H_2_O (0.04); MgSO_4_ *·*7H_2_O (0.075); CaCl_2_ *·*2H_2_O (0.036); ferric citrate (0.006); EDTA (0.001); Na_2_CO_3_ (0.02); citric acid (0.006) and micronutrients. As a carbon source was added to sodium bicarbonate, at concentrations 0.40, 1.00, and 1.60 g L^−1^.

### 2.2. Cultivation Conditions

The cultures were carried out in 6 L open Raceway type bioreactors (0.70 m long, 0.20 m wide, and 0.075 m deep) [12] with 5 L of working volume and initial cellular concentration of 0.20 g L^−1^.

The cultivation stirring was carried out using submersible pumps attached to the bioreactors with a 60 L h^−1^ flow rate. Illumination of 41.6 *μ*mol m^−2^ s^−1^ was provided by daylight type fluorescent light bulbs of 40 w. The experimental apparatus was maintained for 40 d in a thermostatically controlled nonsterile chamber at 30°C with a 12 h light/dark photoperiod [9].

### 2.3. Analytical Determinations

Daily, the biomass concentration (g L^−1^) was determined by measuring the optical density of the liquid medium in a spectrophotometer (FEMTO 700 Plus, Brazil) at 670 nm [13]. The calibration curve has related the optical density with the dry weight of* Chlorella* sp. biomass. The pH was measured every 24 h with a digital pH meter (Quimis Q400H, Brazil).

### 2.4. Studied Responses

The compared responses were the following: specific growth rate (*μ*_mean_), productivity (*P*_mean_), generation time (*t*_*g*_), and produced biomass concentration (*X*_biomass_) when the microalga* Chlorella* sp. was exposed to different concentrations of sodium bicarbonate (0.40, 1.00, and 1.60 g L^−1^), medium renewal rate (30, 40, and 50%), and blend concentration (0.40, 0.60, and 0.80 g L^−1^). The specific growth rate was obtained from

(1) $$\begin{array}{c} \mu_{X}=\frac{1}{X}\frac{dX}{dt}. \end{array}$$

The biomass productivity, defined as the cellular mass formed at a given volume per unit of time, was calculated according to

(2) $$\begin{array}{c} P_{X}=\left(\frac{X-X_{0}}{t-t_{0}}\right), \end{array}$$

where *X* (g L^−1^) is the final cellular concentration, *X*_0_ (g L^−1^) is the initial cellular concentration of the culture, *t*(*d*) is the final time, and *t*_0_ is the initial cultivation time.

The generation time, defined as the time required for the occurrence of doubling of biomass, was obtained from

(3) $$\begin{array}{c} t_{g}=\frac{\ln\left(2\right)}{\mu_{\text{mean}}}. \end{array}$$

The generated biomass, defined as the sum of the concentration of biomass removed after the completion of each growth cycle, was calculated using

(4) Xbiomass =∑XC1−Xi1+XC2−Xi2    +XC3−Xi3⋯+XCn−Xin,

where *X*_*C*1_ (g L^−1^) is the final biomass concentration when the desired blend concentration is obtained in the first cycle, according to the proposed factorial design, *X*_*i*1_ (g L^−1^) is the initial biomass concentration of the cultivation, *X*_*C*2_ (g L^−1^) is the final biomass concentration when the blend concentration is obtained in the second cycle, and *X*_*i*2_ (g L^−1^) is the initial biomass concentration in the cultivation after the blend concentration has been obtained in the first cycle.

### 2.5. Experimental Design

A Box-Behnken factorial design [12] was proposed and modified with three study factors, varying every one in three levels. The change in planning was conducted in the blend concentration, in which the central level was replaced by the higher level and vice versa.  Table 1 shows the matrix of coded variables of the factorial design and the respective levels of the variables.

When the cellular concentration has achieved a predetermined level (0.40, 0.60, or 0.80 g L^−1^ named as “blend concentration”), according to the planning matrix, a withdrawal of 30, 40, or 50% of the medium (medium renewal rate) was carried out and the same fresh medium amount was added. Each experiment lasted 40 d.

The specific growth rate was obtained by exponential regression in the logarithmic phase of multiplication at each time interval corresponding to the respective growth cycle. Biomass productivity, which is defined as the cellular mass formed at a given volume by unit of time, was calculated separately for each cycle. The arithmetic mean and standard deviation were calculated for these responses in each experiment. The concentration of generated biomass was defined as the sum of biomass removed from the culture when the respective blend concentration was achieved.

### 2.6. Statistical Analysis

The arithmetic mean of responses specific growth rate (*μ*_mean_), productivity (*P*_mean_), and produced biomass concentration (*X*_biomass_) was evaluated according to the design of experiment methodology by the main effects and interaction estimation of the blend concentration, sodium bicarbonate concentration, and medium renewal rate. The standard error was calculated by pure error to *μ*_mean_ and *P*_mean_ and residual error to *X*_biomass_, with 90% confidence level.

The studied independent variables influence on *μ*_mean_, *P*_mean_, and *X*_biomass_ was adjusted using a second-degree polynomial function (5), which only considered the regression coefficients that had significant effects on the response:

(5) $$\begin{array}{c} Y=b_{0}+\overset{3}{\underset{i=1}{\sum }}\beta_{i}X_{i}+\overset{3}{\underset{i=1}{\sum }}\beta_{ii}X_{i}^{2}+\overset{2}{\underset{i=1}{\sum }}\overset{3}{\underset{i=1}{\sum }}\beta_{ij}X_{i}X_{j}, \end{array}$$

in which *b*_0_ represents the mean experimental result, *β*_*i*_ are the coefficients of linear regression, *β*_*ii*_ are the coefficients of quadratic regression, and *β*_*ij*_ are the regression coefficients that define the interactions between variables.

## 3. Results and Discussion

The highest specific growth rates (0.149, 0.147, and 0.141 d^−1^) were obtained in experiments 8, 12, and 15 (blend concentrations of 0.60, 0.80, and 0.80 g L^−1^ and medium renewal rates of 40, 50, and 40%, resp.). These results representing an increase of 60% of parameter, when compared with the lowest specific growth rate value, were obtained in experiment 9 (blend concentration of 0.80 g L^−1^, medium renewal rate of 30%, and sodium bicarbonate concentration of 0.40 g L^−1^). The results obtained show that the specific growth rate increased when the concentration of sodium bicarbonate and medium renewal rate were increased, maintaining maximum levels of blend concentration.

According to Vonshak et al. [8] cellular concentrations between 0.40 and 1.00 g L^−1^ for* Spirulina* resulted in a decrease of this microorganism's photosynthetic potential, due to the absence of light incident on most cells. However, concentrations close to 0.50 g L^−1^ are considered ideal for maximum photosynthetic efficiency. Radmann et al. [12] have cultivated* Spirulina platensis* in a semicontinuous mode and they have found that the highest specific growth rates and productivities were obtained when the microalga was exposed to a renewal rate of 40 to 60%. These observations were consistent with the results obtained in this study because the experiment with highest growth rate had a blend concentration of 0.60 g L^−1^ and medium renewal rate of 40%.

The highest productivity was obtained in experiment 11 (0.091 g L^−1^ d^−1^), when the microalga was cultivated with blend concentration of 0.80 g L^−1^, the renewal rate was 30%, and the concentration of sodium bicarbonate was 1.60 g L^−1^, indicating an increase of 190% when compared with the lower values, which were obtained in experiments 1, 5, and 7 (blend concentration of 0.40 g L^−1^ and sodium bicarbonate concentrations of 1.00, 0.40, and 1.60 g L^−1^) (Table 2). The values obtained in assays 1, 5, and 7 were close to those found by Radmann et al. [12] who obtained productivities of 0.027 and 0.039 g L^−1^ d^−1^, when cultivating* Spirulina platensis* in a semicontinuous mode with blend concentrations of 0.40 and 0.80 g L^−1^ and renewal rates of 20 and 40%, respectively. In experiment 11, characterized by the blend concentration of 0.80 g L^−1^, renewal rate of 30%, as well as concentration of sodium bicarbonate of 1.60 g L^−1^, has had the largest number of growth cycles (12 cycles). In this assay 158% more growth cycles were obtained when compared to those obtained in experiments 4 and 6 (Table 2), which have obtained 5 cycles.

The generation time of microalga varied between 4.64 and 7.89 d. Thus, this generation time approached the value obtained by Radmann et al. [12], which was 5.20 d, also in semicontinuous cultivation. At the moment that the cellular duplication speed increases, the generation time decreases, making the cultivation economically viable. Then, smaller generation times are expected. One advantage of growing microalgae is that they can double their biomass in less than a week. Doubling the biomass of terrestrial plants can take months and doubling the concentration of animal proteins can take years [2].

The highest concentrations of generated biomass were obtained in tests 12, 14, and 15, when* Chlorella* sp. was cultivated with blend concentration of 0.80 g L^−1^, medium renewal rate of 50, 40, and 40%, and sodium bicarbonate concentrations of 1.60, 1.00, and 1.00 g L^−1^, respectively. This way, those assays present an increase of 172% when compared to the lower values, which were obtained in experiments 1, 3, 5, and 7 when the microalgae were cultivated with the blend concentration of 0.40 g L^−1^, regardless of the medium renewal rate and concentration of sodium bicarbonate used (Table 2).

**Figure 1:**
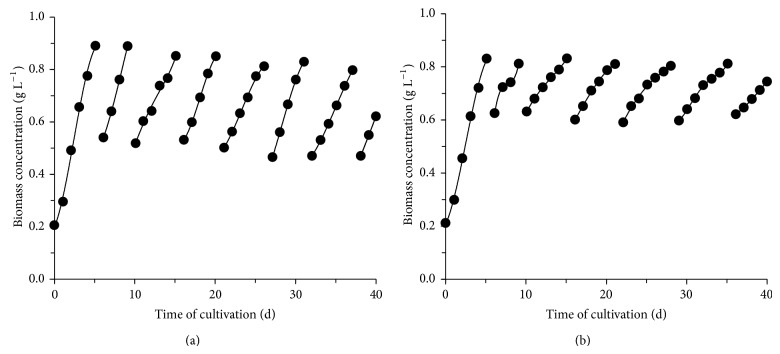
Cellular concentration of semicontinuous cultivation as a function of time for (a) blend concentration of 0.80 g L^−1^, medium renewal rate of 50%, and sodium bicarbonate concentration of 1.60 g L^−1^ (experiment  12) and (b) blend concentration of 0.80 g L^−1^, medium renewal rate of 30%, and sodium bicarbonate concentration of 0.40 g L^−1^ (experiment  9).

Figure 1 shows the better behavior characteristic of semicontinuous cultivation among the assays of this work, with the growth profile of the assays 12 and 9 (blend concentration of 0.80 g L^−1^, medium renewal rate of 50 and 30%, and sodium bicarbonate concentration of 1.60 and 0.40 g L^−1^, resp.). In assay 12, the microorganism has had one of the highest productivities (0.087 g L^−1^ d^−1^) and a high and relatively constant specific growth rate throughout the process (0.147 ± 0.026 d^−1^). In culture 9, it can be seen that as the medium renewals were carried out (cycles), there was a reduction in the specific growth rate, from 0.275 d^−1^ in the first cycle to 0.050 d^−1^ at the last cycle. As this medium has had a lower level sodium bicarbonate concentration, the dilution of this nutrient may have become severe, reducing the rate of cellular multiplication. When the blend concentrations are carried out, toxic substances produced from the microorganism or from the culture medium end up hindering its development in the medium [14]. Thus, it had better to stop the semicontinuous cultivation once the ideal conditions of cultivation are no longer present.

The* Chlorella* sp. semicontinuous cultivation shows that the specific growth rate of the microalga was increased when the medium renewal rate increased. The authors have also found that, with renewal rates of 40 to 50%, the percentage of conversion of nitrogen in the cells was below 100%, indicating that there was no nitrogen limitation [10].

**Figure 2:**
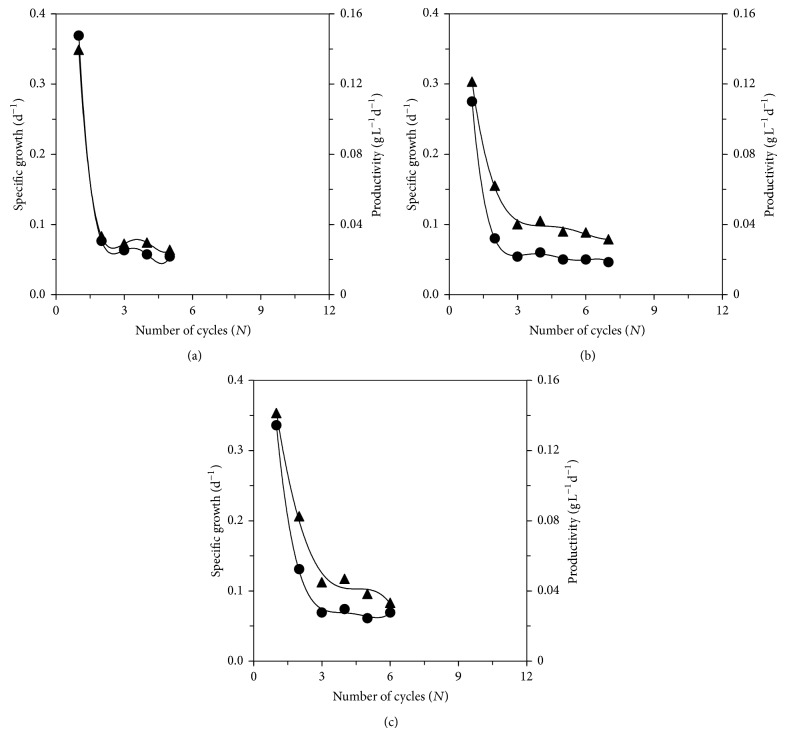
Evolution of the specific growth rate (●) and productivity (▲) as a function of number of cycles for (a) blend concentration of 0.60 g L^−1^, medium renewal rate of 40%, and sodium bicarbonate concentration of 0.40 g L^−1^ (experiment  6); (b) blend concentration of 0.80 g L^−1^, medium renewal rate of 30%, and sodium bicarbonate concentration of 0.40 g L^−1^ (experiment  9); and (c) blend concentration of 0.80 g L^−1^, medium renewal rate of 50%, and sodium bicarbonate concentration of 0.40 g L^−1^ (experiment  10).

Figure 2 shows the behavior of the specific growth rate and productivity as a function of the number of cycles for experiments 6, 9, and 10 (blend concentrations of 0.60, 0.80, and 0.80 g L^−1^, renewal rates of 40, 30, and 50%, and sodium bicarbonate concentrations of 0.40 g L^−1^, resp.). For the 3 experiments, the highest rates (0.369, 0.275, and 0.336 d^−1^, resp.) and productivities (0.139, 0.129, and 0.144 g L^−1^ d^−1^, resp.) were obtained in the first cycle of growth and have considerably decreased in the following cycle, after which they have remained approximately constant until the end of cultivation.

The limited growth in dense cultures may occur due to shading caused by the cells themselves while they grow, preventing part of the culture from receiving light incidence. Photolimitation is one of the main problems in the cultivation of microalgae, which occurs because the cells on the surface create shade for those at greater depths within the culture medium [15, 16]. Vonshak et al. [8] found that 0.40 to 0.50 g L^−1^ was the optimal cellular concentration for maximum photosynthetic efficiency in* Spirulina* cultures, and at this concentration it is estimated that at 20–30 mm from the surface about 80% of the cells are exposed to complete darkness at certain moments.

Radmann et al. [12] have assessed the semicontinuous cultivation of* Spirulina* in Raceway bioreactor, where the highest specific growth rates were 0.134 d^−1^ and 0.138 d^−1^ when the microalgae were cultivated in standard Zarrouk medium and 20% diluted Zarrouk medium, respectively, using renewal rates of 40 and 60% over a period of 62 d of cultivation. Costa et al. [3] used a natural culture medium, with water from Mangueira Lagoon supplemented with bicarbonate and urea, for the discontinuous cultivation of* Spirulina platensis* in open bioreactor, and obtained a maximum specific growth rate of 0.157 d^−1^, which is a value that is higher than those ones obtained in semicontinuous cultivation. However, the exponential growth phase has lasted only 15 d, whereas, in the semicontinuous cultivation of this study, the specific growth rate remained at around 0.10 d^−1^ for 40 d of cultivation, which shows the usefulness of this mode of cultivation.

Henrard et al. [17] have cultivated* Cyanobium* sp. in closed tubular photobioreactors and have observed that, in cultures with low cellular concentrations, there was an inhibitory effect for the doubling of microalga biomass. According to Vonshak [18], photoinhibition is the reduction of photosynthetic capacity due to damage caused by light that is more intense than that one required for photosynthesis. The highest productivities obtained by Henrard et al. [17] were when the microalga had grown at the highest blend concentrations (1.00 g L^−1^ and 1.20 g L^−1^) and maximum medium renewal rates (50%) and also had the largest number of cycles (10 cycles) during the cultivation.

The effects and the significance levels obtained from Box-Behnken factorial design analysis used in the semicontinuous cultivation are presented in Table 3. The linear significant effects were achieved by the blend concentration and medium renewal rate, while a quadratic effect was accomplished only by the medium renewal rate, both significant at *P* < 0.10 to the specific growth rate (Table 3). Regarding the effect of blend concentration on the specific growth rate, it was positively affected with an increase of 0.016 d^−1^ in the response when there was an increase of 0.40 to 0.80 g L^−1^ in blend concentration used. The interaction between blend concentration and the sodium bicarbonate (*X*_1_(*L*)*·X*_3_(*L*)) has also shown significant positive effect (*P* < 0.10); that is, increasing concentrations of *X*_1_ and *X*_3_ generates an increase in the specific growth rate of 0.029 d^−1^.

For productivity, the effects of linear blend concentration and sodium bicarbonate concentration were significant (*P* < 0.10) and positive, indicating that increasing these concentrations generated an increase of 0.042 and 0.014 g L^−1^ d^−1^ in the productivity, respectively. The effect of interaction between blend concentration and sodium bicarbonate concentration (*X*_1_(*L*)*·X*_3_(*L*)) was significant (*P* < 0.10) and positive, increasing productivity in 0.018 g L^−1^ d^−1^ when both variables were increased from 0.4 to 0.8 g L^−1^ and from 0.4 to 1.6 g L^−1^, respectively.

For biomass production obtained, the linear effects, *X*_1_(*L*), *X*_2_(*L*), and *X*_3_(*L*), quadratic effect *X*_3_(*Q*), and interaction effect between *X*_1_(*L*) and *X*_3_(*L*) were significant (*P* < 0.10) and positive. However, the linear effect of blend concentration had greater module than the other effects, producing a greater influence and increasing the biomass production in 1.430 g L^−1^.

The models presented in (6), (7), and (8) were obtained from the statistical analysis of the Box-Behnken factorial design and predict *μ*_mean_, *P*_mean_, and *X*_biomass_ as a function of blend concentration (*X*_1_), medium renewal rate (*X*_2_), and sodium bicarbonate concentration (*X*_3_) for the semicontinuous cultivation* Chlorella* sp. These models are useful to optimize the production of microalga biomass because the levels of the variables tested may be selected so that the optimum is obtained.

**Figure 3:**
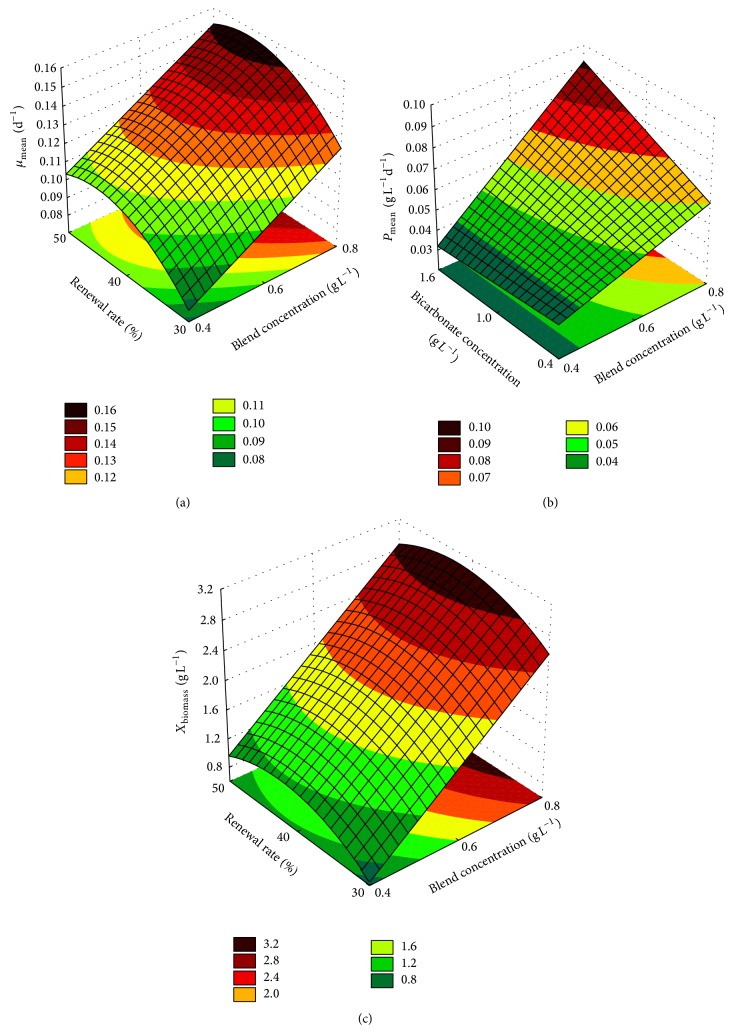
Response surfaces and contour lines for (a) specific rate as a function of blend concentration and medium renewal rate using 1.60 g L^−1^ of sodium bicarbonate concentration; (b) productivity as a function of the sodium bicarbonate concentration and blend concentration using a medium renewal rate of 40%; and (c) generated biomass concentration during cultivation as a function of the medium renewal rate and blend concentration using sodium bicarbonate concentration of 1.60 g L^−1^.

The correlation coefficients (*R*) for the simulation of the response surface equations are 0.886, 0.950, and 0.977 for (6), (7), and (8), respectively. The model obtained based on these equations is represented by the response surfaces shown in Figure 3. Consider

(6) μmean=0.128+0.008X1+0.013X2−0.015X22+0.016X1X3,

(7) $$\begin{array}{c} P_{\text{mean}}=0.055+0.021X_{1}+0.007X_{3}+0.009X_{1}X_{3}, \end{array}$$

(8) Xbiomass=1.89+0.725X1+0.154X2−0.327X22+0.192X3+0.229X1X3.

Figures 3(a) and 3(c) show the response surfaces for the specific growth rate (*μ*_mean_) and produced biomass concentration (*X*_biomass_) as a function of blend concentration and medium renewal rate using a sodium bicarbonate concentration of 1.60 g L^−1^, while Figure 3(b) shows the response surfaces for the productivity (*P*_mean_) as a function of blend concentration and sodium bicarbonate concentration using a renewal rate of 40%. The maximum specific growth rates (Figure 3(a)) were obtained with a blend concentration of 0.80 g L^−1^, medium renewal rate of 40%, and sodium bicarbonate concentration of 1.60 g L^−1^. The lowest specific rates were found at minimum concentrations of blend concentration and medium renewal rate, utilizing also 1.60 g L^−1^ sodium bicarbonate concentration.

The highest productivity (Figure 3(b)) was obtained with a blend concentration of 0.8 g L^−1^, sodium bicarbonate concentration of 1.6 g L^−1^, and medium renewal rate of 40%. As regards generated biomass during cultivation, the maximum concentrations of biomass were obtained when the microalga had been cultivated with a blend concentration of 0.80 g L^−1^, medium renewal rate of 40%, and sodium bicarbonate concentration of 1.60 g L^−1^. The lowest concentrations of biomass (Figure 3(c)) were obtained when the microalga had grown at minimum blend concentrations (0.40 g L^−1^) and renewal rate conditions (30%), utilizing sodium bicarbonate concentrations 1.60 g L^−1^.

The carbon feeding concentration is an important factor for the microalgae cultivation. This fact is very important because the biomass microalgae, according to Amaro et al. [19], have around 50% w/w of the element in its composition. This fact is justified because carbon composes large amount of molecules organicly synthesized by cells, such as proteins, carbohydrates, and lipids. Air contains 0.038% of CO_2_, so the addition of CO_2_ or another form of carbon to the culture is required to achieve higher maximum specific growth and productivities.

Radmann et al. [12] have obtained a specific growth rate 0.138 d^−1^ cultivating* Spirulina* with blend concentration of 0.40 g L^−1^, 40% renewal rate of diluted Zarrouk medium in distilled water, and the blend concentration of 0.60 g L^−1^ and concentration of Zarrouk to 50% and reduction of renewal rate to 20% decreased the specific growth rate to 0.038 d^−1^. The results are partially consistent with those obtained in this study, because as the medium renewal rate was decreased from 40% and 50% to 30% and the concentration of sodium bicarbonate from 1.60 g L^−1^ to 0.40 g L^−1^, there was a decrease in the specific growth rate from 0.149 d^−1^ to 0.087 d^−1^.

Reichert et al. [9] have studied the semicontinuous cultivation of* Spirulina platensis* with Zarrouk medium in a closed photobioreactor and obtained a maximum productivity, around of 0.04 g L^−1^ d^−1^, with blend concentration being 0.50 g L^−1^ and the medium renewal rate being 25% and 50%. The data obtained in this study were higher than those obtained by Reichert et al. [9], where the highest productivities of 0.091 g L^−1^ d^−1^ and 0.087 g L^−1^ d^−1^ were obtained with medium renewal rates of 30% and 50% but with a blend concentration of 0.80 g L^−1^.

## 4. Conclusion

The present experiments have shown that the growth of* Chlorella* sp. is major when it is subjected to a higher concentration of sodium bicarbonate. Thus, with 1.6 g L^−1^ of carbon source, 40% of renewal rate, and 0.8 g L^−1^ of blend concentration are obtained the higher results for specific growth rate, biomass generated, and biomass productivity for* Chlorella* sp.

## Figures and Tables

**Table 1:** Matrix of the Box-Behnken type factorial design with the coded variables and their respective levels of variation.

Experiment	*X*_1_	*X*_2_	*X*_3_	*X*_1_ (g L^−1^)	*X*_2_ (%)	*X*_3_ (g L^−1^)
1	−1	−1	0	0.40	30	1.00
2	0	−1	0	0.60	30	1.00
3	−1	+1	0	0.40	50	1.00
4	0	+1	0	0.60	50	1.00
5	−1	0	−1	0.40	40	0.40
6	0	0	−1	0.60	40	0.40
7	−1	0	+1	0.40	40	1.60
8	0	0	+1	0.60	40	1.60
9	+1	−1	−1	0.80	30	0.40
10	+1	+1	−1	0.80	50	0.40
11	+1	−1	+1	0.80	30	1.60
12	+1	+1	+1	0.80	50	1.60
13	+1	0	0	0.80	40	1.00
14	+1	0	0	0.80	40	1.00
15	+1	0	0	0.80	40	1.00

*X*_1_: blend concentration, *X*_2_: medium renovation rate, and *X*_3_: concentration of sodium bicarbonate.

**Table 2:** Growth cycles (*N*), mean specific growth rate (*μ*_mean_), productivity (*P*_mean_), generation time (mean *t*_*g*_), and concentration of biomass produced (*X*_biomass_) for the *Chlorella* sp. microalgae cultivated in bioreactors open in semicontinuous mode.

Experiment	*N*	*µ*_mean_ (d^−1^)	*P*_mean_ (g L^−1^ d^−1^)	*t*_*g* mean_ (d)	*X*_biomass_ (g L^−1^)
1	8	0.099 ± 0.122	0.033 ± 0.036	6.98 ± 0.12	0.86
2	6	0.090 ± 0.092	0.040 ± 0.032	7.64 ± 0.09	1.28
3	6	0.130 ± 0.114	0.041 ± 0.034	5.32 ± 0.11	1.12
4	5	0.117 ± 0.054	0.050 ± 0.018	5.90 ± 0.05	1.63
5	7	0.126 ± 0.012	0.038 ± 0.003	5.48 ± 0.01	1.28
6	5	0.124 ± 0.137	0.051 ± 0.049	5.58 ± 0.13	1.37
7	6	0.100 ± 0.018	0.031 ± 0.006	6.91 ± 0.01	1.04
8	10	0.149 ± 0.076	0.069 ± 0.029	4.64 ± 0.07	2.08
9	7	0.087 ± 0.083	0.052 ± 0.031	7.89 ± 0.08	1.76
10	6	0.123 ± 0.107	0.064 ± 0.041	5.62 ± 0.10	2.02
11	12	0.134 ± 0.081	0.091 ± 0.037	5.57 ± 0.09	2.47
12	8	0.147 ± 0.065	0.087 ± 0.026	4.71 ± 0.06	2.84
13	7	0.128 ± 0.041	0.075 ± 0.015	5.41 ± 0.04	2.51
14	8	0.139 ± 0.036	0.084 ± 0.012	4.97 ± 0.03	2.89
15	8	0.141 ± 0.050	0.085 ± 0.016	4.89 ± 0.05	2.80

**Table 3:** Statistical significances (*p*) and effects obtained from the analysis of the factorial design used in the semicontinuous cultivation of *Chlorella *sp.

Factor	*µ*_mean_ (d^−1^)			*P*_mean_ (g L^−1^ d^−1^)			*X*_biomass_ (g L^−1^)		
	Effect	S. e.^*^	*p*	Effect	S. e.^*^	*p*	Effect	S. e.^*^	*p*
Mean	0.119	0.002	<0.001	0.054	0.002	0.001	1.647	0.051	<0.001
*X*_1_(*L*)	0.016	0.004	0.071	0.042	0.003	0.007	1.430	0.111	<0.001
*X*_1_(*Q*)	−0.002	0.004	0.742	−0.004	0.003	0.336	−0.200	0.104	0.114
*X*_2_(*L*)	0.028	0.005	0.033	0.007	0.004	0.223	0.302	0.131	0.069
*X*_2_(*Q*)	0.015	0.004	0.058	0.007	0.003	0.133	0.342	0.092	0.014
*X*_3_(*L*)	0.010	0.005	0.185	0.014	0.004	0.079	0.384	0.131	0.032
*X*_3_(*Q*)	−0.001	0.004	0.764	0.001	0.003	0.808	0.119	0.092	0.253
*X*_1_(*L*)*·X*_2_(*L*)	−0.003	0.006	0.647	−0.002	0.005	0.665	0.024	0.151	0.881
*X*_1_(*L*)*·X*_3_(*L*)	0.029	0.006	0.040	0.018	0.005	0.059	0.458	0.151	0.029
X_2_(*L*)*·*X_3_(*L*)	−0.012	0.007	0.242	−0.008	0.006	0.284	0.054	0.177	0.772

*X*_1_: blend concentration; *X*_2_: medium renewal rate; *X*_3_: concentration of sodium bicarbonate; *L*: linear effect; *Q*: quadratic effect; *µ*_mean_: mean specific growth rate; *P*_mean_: mean productivity; *X*_biomass_: concentration of generated biomass during cultivation.

^*^S. e.: standard error.
